# PAX*2*^+^ Mesenchymal Origin of Gonadal Supporting Cells Is Conserved in Birds

**DOI:** 10.3389/fcell.2021.735203

**Published:** 2021-08-27

**Authors:** Martin A. Estermann, Mylene M. Mariette, Julie L. M. Moreau, Alexander N. Combes, Craig A. Smith

**Affiliations:** ^1^Department of Anatomy and Developmental Biology, Monash Biomedicine Discovery Institute, Monash University, Clayton, VIC, Australia; ^2^Centre for Integrative Ecology, School of Life and Environmental Sciences, Deakin University, Geelong, VIC, Australia

**Keywords:** PAX2, sex determination, Evo-Devo, gonadal sex differentiation, DMRT1, embryonic gonad

## Abstract

During embryonic gonadal development, the supporting cell lineage is the first cell type to differentiate, giving rise to Sertoli cells in the testis and pre-granulosa cells in the ovary. These cells are thought to direct other gonadal cell lineages down the testis or ovarian pathways, including the germline. Recent research has shown that, in contrast to mouse, chicken gonadal supporting cells derive from a *PAX2/OSR1/DMRT1/WNT4* positive mesenchymal cell population. These cells colonize the undifferentiated genital ridge during early gonadogenesis, around the time that germ cells migrate into the gonad. During the process of somatic gonadal sex differentiation, PAX2 expression is down-regulated in embryonic chicken gonads just prior to up-regulation of testis- and ovary-specific markers and prior to germ cell differentiation. Most research on avian gonadal development has focused on the chicken model, and related species from the Galloanserae clade. There is a lack of knowledge on gonadal sex differentiation in other avian lineages. Comparative analysis in birds is required to fully understand the mechanisms of avian sex determination and gonadal differentiation. Here we report the first comparative molecular characterization of gonadal supporting cell differentiation in birds from each of the three main clades, Galloanserae (chicken and quail), Neoaves (zebra finch) and Palaeognathe (emu). Our analysis reveals conservation of PAX2^+^ expression and a mesenchymal origin of supporting cells in each clade. Moreover, down-regulation of PAX2 expression precisely defines the onset of gonadal sex differentiation in each species. Altogether, these results indicate that gonadal morphogenesis is conserved among the major bird clades.

## Introduction

Gonadal sex differentiation during embryogenesis provides an excellent model for studying the genetic regulation of development. The somatic component of the vertebrate gonad arises from intermediate mesoderm, while the germ cells are of extra-gonadal origin, migrating into the gonad before somatic sex differentiation commences (Lawson, 1999; Nef et al., 2019). Among most vertebrates, the gonadal primordium, together with its germ cells, is initially morphically identical in both sexes. Subsequently, the somatic cells and the germ cells of the gonad are directed down the testicular or ovarian pathway via a cascade of sexually dimorphic gene expression that starts in the somatic compartment (Eggers et al., 2014; Liu et al., 2016; Yang et al., 2018). Two distinctive structures are initially distinguishable in the somatic compartment, an outer coelomic epithelium and underlying medulla (Smith and Sinclair, 2004). In the mouse, for which most data are available, proliferation of cells in the coelomic epithelium gives rise to so-called supporting cell progenitors, which enter the medulla. This key cell lineage generates Sertoli cells in the testis and pre-granulosa cells in the ovary. In both sexes, the supporting cells are thought to direct other uncommitted progenitor cells to the testicular or ovarian pathways, including the germline (Piprek et al., 2017; Rotgers et al., 2018). Germ cells are specified in the epiblast very early in development, and they migrate into the undifferentiated gonads (via the hindgut in mammals, via the bloodstream in birds) (Tagami et al., 2017). The germ cells of both sexes populate the gonad but are uncommitted to either the spermatogenesis or oogenesis pathway until somatic gonadal cells send inductive cues. Germ cell fate is therefore closely linked to somatic development of the gonad. In mouse, after the germ cells have settled in the gonads, the somatic supporting cell lineage begins to differentiate. In female mammals, somatic, and intrinsic signals induce germ cells to express *Stra8* and enter meiosis during embryogenesis. A large body of evidence previously pointed to retinoic acid (RA) as the somatic indicative signal triggering meiosis in females (Bowles et al., 2006; Koubova et al., 2006). However, surprisingly, recent data has shown that compound mutant mouse ovaries lacking all retinoic acid receptors or all three RALDH2 enzymes that synthesize RA can still initiate meiosis (Chassot et al., 2020; Vernet et al., 2020). The exact gonadal somatic signal for female germ cell sexual development is therefore again open to investigation. In male mammal gonads, the germ cells do not enter meiosis during embryogenesis. Instead, they lose pluripotency and enter mitotic arrest (Spiller et al., 2017). These sexually dimorphic germ cell fates are intimately linked to the development of the gonadal somatic cells. In males, this lineage gives rise to pre-Sertoli cells and, in females, pre-granulosa cells (Stevant and Nef, 2019). Signals such as Fgf9 sent from the Sertoli cells act with intrinsic factors, such as Nanos2, to antagonize meiosis and instead direct the germ cells down the male pathway, toward spermatogenesis (Suzuki and Saga, 2008; Bowles et al., 2010). The supporting cell lineage also sends inductive signals to the presumptive steroidogenic lineage, directing their differentiation into Leydig cells (in the testis) or thecal cells (in the ovary) (Yao et al., 2002; Rebourcet et al., 2014). In the developing mammalian ovary, proper follicle formation requires cross-talk between the female somatic and germ cell populations (Liu et al., 2010; Baillet and Mandon-Pepin, 2012). Hence, the sexual fate of the gonadal soma and the germ cells hinges upon the key supporting cell lineage.

In the mouse, the key supporting cell lineage derives from the coelomic epithelium via asymmetric cell division and egression into the underlying gonadal mesenchyme (Piprek et al., 2016; Lin et al., 2017; Stevant and Nef, 2019). Surprisingly, recent research has shown that, in contrast to mammals, the coelomic epithelium in the chicken embryo does not generate the supporting cell lineage (Sertoli or pre-granulosa cells). Rather, it gives rise to a non-steroidogenic interstitial cell population (Estermann et al., 2020). In chicken, the supporting cells develop from a mesenchymal source present in the gonad during early development (Sekido and Lovell-Badge, 2007; Estermann et al., 2020). These cells have a specific molecular signature, expressing the transcription factors *PAX2, DMRT1* and *OSR1*, and the signaling molecule, *WNT4*. The finding that supporting cells in chicken derive form a different source to those in mouse was surprising, given the conservation of overall gonadal morphogenesis among vertebrate embryos (DeFalco and Capel, 2009). However, a major difference between birds and mammals is the genetic gonadal sex-determining trigger. In mouse and other mammals, the Y chromosome-linked *SRY* gene operates as the master sex switch, initiating Sertoli cell differentiation in male embryos (Koopman et al., 1990, 1991; Sinclair et al., 1990; Goodfellow and Lovell-Badge, 1993). *SRY* is absent outside the mammalian clade, and in fact, birds have a different sex chromosome system. Birds have ZZ/ZW sex chromosomes, in which male (ZZ) is the homogametic sex and female (ZW) is heterogametic (Marshall Graves, 2008). The Z linked gene, *DMRT1* operates as the testis determining factor via a dosage mechanisms (Smith et al., 2009; Lambeth et al., 2014; Ioannidis et al., 2021). Due to the lack of Z sex chromosome compensation, male supporting cells have double the dose of *DMRT1* compared to females (Raymond et al., 1999; Smith et al., 2003; Ayers et al., 2015). *DMRT1* knockdown or knock out results in feminization of the gonad. Moreover, over-expression of this gene causes gonadal masculinization, indicating that *DMRT1* is the sex-determining gene in chicken, and presumably in all birds (Smith et al., 2009; Lambeth et al., 2014; Ioannidis et al., 2021). This would be consistent with the deep evolutionary conservation of the Z sex chromosome in birds, across some 60 million years (Handley et al., 2004; Zhou et al., 2014; Xu et al., 2019). In the male chicken embryo, DMRT1 is known to activate *SOX9* expression, which is crucial in Sertoli differentiation, and AMH, which is important for Müllerian duct regression (Lambeth et al., 2014). In females (ZW), due to the lower levels of *DMRT1* expression, supporting cells differentiate toward pre-granulosa cells by upregulating FOXL2 and Aromatase (Lambeth et al., 2013; Major et al., 2019). We previously characterized cell lineage specification during chicken gonadal sex differentiation and identified *PAX2* as a novel marker of the early supporting cell lineage (Estermann et al., 2020). During the process of gonadal sex differentiation, *PAX2* expression is down-regulated in chicken gonads (Estermann et al., 2020). This suggests that PAX2 down-regulation could be used to predict the onset of gonadal sex differentiation in chicken. However, the conservation of both the mesenchymal origin of gonadal cells and the role of PAX2 in birds beyond the chicken have not been previously explored.

Modern birds are classified into two main groups, the Palaeognathe, (the flightless ratites and volant tinamous) and Neognathae (all other birds). The Neognathae is divided into two clades, the Galloanserae (chickens, quails, and ducks et al.) and Neoaves, the perching birds (around 95% of all extant avian species) (Cracraft, 2001; Hackett et al., 2008). Most research on avian gonadal development has focused on the chicken, or related members of the Galloanserae clade, such as quail and duck (Takada et al., 2006; Smith et al., 2009; Ayers et al., 2015; Bai et al., 2020; Okuno et al., 2020). Additionally, these studies have focused mainly on the conservation of mammalian genes involved in gonadal sex differentiation. There is very little information regarding gonadal sex differentiation among the other major avian clades (Hirst et al., 2017a). Comparative analysis in birds is required to fully understand the mechanism of avian sex determination and gonadal sex differentiation.

Historically, gonadal sex differentiation has been characterized on the basis of morphology, whereby the condensation of Sertoli cells marks the onset of testis formation and organization of pre-follicular cells marks the onset of ovary formation (Wilhelm et al., 2007). In the chicken embryo, the first morphological sign of testis formation, as in mammals, is the appearance of Sertoli cells and their coalescence in the medullary cords of the gonad. In the female chicken embryo, the first overt morphological sign of sex differentiation is a thickening of the outer coelomic epithelium into a cortex and fragmentation of the underling medulla (Carlon and Stahl, 1985). However, histology alone has proven to be inconsistent and inaccurate to determine the precise time of gonadal sex differentiation onset. A clear example is studies on Japanese quail embryos (*Coturnix japonica*). Several histological hematoxylin-eosin based analyses determined that quail gonads were sexually differentiated at embryonic day 6 (E6) (stage 30), E7 (stage 32) or E8 (stage 35) (Kannankeril and Domm, 1968; Intarapat and Satayalai, 2014; Mohamed et al., 2017). However, sexual differentiation is likely to be triggered at the genetic level prior to overt histological differentiation. In the quail, the Sertoli cell marker *SOX9* is detectable at E5 (stage 27) (Takada et al., 2006), indicating that sexual differentiation begins at the molecular level distinctly prior to morphological differentiation. The development of more accurate molecular methods to determine sexual differentiation is required to improve knowledge of avian sex determination and for informing methodologies targeting species conservation.

Here we report the first comparative molecular characterization of gonadal sex differentiation in birds from each of the three main clades, Galloanserae (chicken and quail), Palaeognathe (emu), and Neoaves (zebra finch). Our analysis demonstrates a conservation of the PAX2^+^ mesenchymal origin of supporting cells in all analyzed birds. In addition, PAX2 down-regulation immediately precedes up-regulation of male and female supporting cell markers, and the morphological onset of sexual differentiation. PAX2 gonadal down-regulation precisely predicted the onset of sex differentiation in each avian clade, more accurately than previous histological analysis. Altogether these results indicate that the process of gonadal sex differentiation is conserved among the major bird clades. This research proposes PAX2 immunodetection as a new methodology to evaluate gonadal differentiation in birds.

## Materials and Methods

### Eggs

Fertilized HyLine Brown chicken eggs (*Gallus gallus domesticus*) were obtained from Research Poultry Farm (Victoria, Australia). Wild type Japanese quail eggs (*Coturnix japonica*) were provided by the Monash transgenic quail facility. Fertilized emu (*Dromaius novaehollandiae*) eggs were purchased from Emu Logic (Toorahweenah, NSW). Zebra finch (*Taeniopygia guttata*) embryos were obtained from wild-derived birds. The zebra finch colony is a captive population derived from wild caught birds under Deakin University Animal Ethics #G23-2018. The birds used in this study were several generation-captive birds derived from this initial population. Fresh eggs were collected in nests in outdoor aviaries and artificially incubated at Deakin University (Geelong, Australia). Eggs were incubated under humid conditions at 37.5°C until collection and staged (Hamburger and Hamilton, 1951; Ainsworth et al., 2010; Nagai et al., 2011; Murray et al., 2013).

### Sexing PCR

A small piece of limb tissue was digested in 30 μl of PCR compatible digestion buffer (10 mM Tris-HCL (pH8.3); 50 mM KCl; 0.1 mg/mL gelatin; 0.45% NP-40; 0.45% Tween-20 containing Proteinase K at 200 μg/mL) and incubated for 20 min at 55°C followed by 6 min at 95°C and hold at 4°C (Clinton et al., 2001). Chicken sexing PCR was performed as previously described (Clinton et al., 2001; Hirst et al., 2017a).

The quail PCR sexing protocol is a modification of a previously described method (Dickens et al., 2012). This method relies upon specific amplification of a female (W) restricted sequence called *WPKCI*. The reaction was performed in a final volume of 10μL containing 1x Go-Taq buffer (Promega), 1.5 mM MgCl2, 0.2 mM dNTP’s, 0.5 μM of each *18S* rRNA primers (forward: 5′-*AGCTCTTTCTCGATTCCGTG*-3′; reverse: 5′-*GGGTAGACACAAGCTGAGCC*-3′) 1μM of each *qWPKCI* primers (forward: 5′-*TTGGGCATTTGAAGATTGT*C-3′; reverse: 5′-*GTCTGAAGGGTCTGAGGGT-*3′), 0.5U Go Taq polymerase (Promega) and 1 μL of the tissue digestion. The PCR program consisted of denaturation for 2 min at 94°C followed by 25 cycles of incubation at 94°C × 10 s; 56°C × 10 s; 72°C × 10 s and final extension at 72°C for 5 min, followed by 4°C hold.

Emu sexing PCR protocol is a modification of a previously described method (Huynen et al., 2002). This method relies upon sex-specific amplification of a W-linked (female) DNA fragment. The sexing reaction was performed in a final volume of 20 μL containing 1x Go-Taq buffer (Promega), 1.5 mM MgCl2, 0.2 mM dNTP’s, 0.5 μM of each sexing primers (forward: 5′-*CCTTTAAACAAGCTRTTAAAGCA*-3′; reverse: 5′-*TCTCTTTTGTTCTAGACAMCCTGA*-3′), 0.5U Go Taq polymerase (Promega) and 1 μL of the tissue digestion. The PCR program consisted of denaturation for 2 min at 95°C followed by 10 cycles of incubation at 95°C × 15 s; 55°C × 20 s; 72°C × 20 s, 25 cycles of incubation at 95°C × 15 s; 47°C × 20 s; 72°C × 20 s and final extension at 72°C for 7 min, followed by 4°C hold.

Zebra finch sexing PCR protocol is a modification of a previously described method (Soderstrom et al., 2007). This method relies upon specific amplification of a fragment of the *CDH* gene located on the W chromosome. As an internal control, a fragment of the *CHD* gene located in the Z chromosome was amplified. The reaction was performed in a final volume of 11 μL. W1 (5′-*GGGTTTTGACTGACTAACTGAT*T-3′), W2 (5′-*GTTCAAAGCTACATGAATAAACA*-3′), Z1 (5′-*GTGTAGTCCGCTGCTTTTGG*-3′) and Z2 (5′-*GTTCGTGGTCTTCCACGTTT*-3′) primers used at a final concentration of 0.1 μM each. 1 μL of digestion buffer was used with 10 μL of the sexing mix. The PCR program consisted of denaturation for 2 min at 94°C followed by 30 cycles of incubation at 94°C × 30 s; 56°C × 45 s; 72°C × 45 s and final extension at 72°C for 5 min, followed by 4°C hold. PCR products and molecular ladder (1 kb plus, Invitrogen) were run on a 2% agarose gel for 30 min at 130 V and visualized with gel red (Biotium).

### Immunofluorescence

Whole embryos or urogenital systems were collected, briefly fixed in 4% PFA/PBS for 15 min, cryo-protected in 30% sucrose overnight and blocked in OCT embedding compound for sectioning. Immunofluorescence was carried out as described previously (Estermann et al., 2020). Briefly, 10 μm frozen sections were cut and antigen retrieval was performed for DMRT1 and PAX2 protein immunofluorescence using the Dako PT Link automated system. Sections were then permeabilized in 1% Triton X-100 in PBS for 10 min at room temperature and washed 3 times in PBS. All sections were blocked in 2% BSA in PBS for 1 h at room temperature followed by primary antibody incubation overnight at 4°C in 1% BSA in PBS. The following primary antibodies were used: rabbit anti-PAX2 (Biolegend 901001, 1;500), rabbit anti-DMRT1 (in house antibody; 1:2000), rabbit anti-AMH (Abexa ABX132175; 1:1000), rabbit anti-Aromatase (in house antibody; 1:4000), rabbit anti-FOXL2 (in house antibody; 1:2000), and rabbit anti-SOX9 (Millipore AB5535, 1:4000). After overnight incubation with primary antibody, sections were then washed 3 times in PBS and incubated for 1 h at room temperature with Alexa Fluor 488 donkey anti-Rabbit (1:1000) and Alexa Fluor 594 donkey anti-Mouse (1:1000) in 1% BSA in PBS. Sections were counterstained in DAPI/PBS and mounted in Fluorsave (Millipore). Images were collected on a Zeiss Axiocam MRC5 microscope using the same exposure time between males and females for expression comparisons.

For double immunofluorescence using two primary antibodies raised in the same species (rabbit anti-PAX2 and rabbit anti-DMRT1), the iterative indirect immunofluorescence imaging (4i) protocol was used on paraffin sections (Gut et al., 2018). Dissected gonads were fixed overnight in 4% paraformaldehyde at 4°C, paraffin-embedded and sectioned in the transverse plane at 5μm. After deparaffinisation, antigen retrieval was carried out using TE buffer (Moreau et al., 2019). Sections were incubated with anti-DMRT1 antibody (1:2000, in house) and anti-cytokeratin antibody (1:200, Novus Bio NBP2-29429) overnight at 4°C. Sections were then washed with 1X PBS and incubated with Donkey anti-Rabbit Alexa Fluor^®^ Plus 647 (1:2000, Invitrogen) and Donkey anti-mouse Alexa Fluor^®^ Plus 488 (1:2000, Invitrogen) together with DAPI for 2 h at room temperature, after which the tissue was washed with 1X PBS. Slides were mounted in imaging buffer (Gut et al., 2018) and images were captured using a 3i Marianas spinning disk confocal at low laser power. Sections were washed in 1X PBS and antibodies were eluted following the 4i protocol. After elution, sections were imaged again with the same parameters to ensure that the first round of antibody labels were removed. Slides were then incubated with anti-PAX2 (1:400, Biolegend 901001) and anti-cytokeratin (1:200, Novus Bio NBP2-29429) in 5% BSA in 1X PBS overnight at 4°C with the same wash, secondary antibody incubation and imaging parameters from the first round of labeling. Brightness and contrast were equally altered across all images to improve data display using ImageJ (Schneider et al., 2012).

### qRT-PCR

Gonadal pairs were collected in Trizol reagent (Sigma-Aldrich) homogenized and Phenol-Chloroform RNA extraction was performed as per the manufacturer’s instructions (Trizol, Invitrogen). DNA-free^TM^ DNA Removal Kit (Invitrogen) was used to remove genomic DNA. 100-500 ug of RNA was converted into cDNA using Promega Reverse Transcription System. QuantiNova SYBR^®^ Green PCR Kit was used to perform qRT-PCR. PAX2 expression levels were quantified by Pfaffl method (Pfaffl, 2001) using β-actin as internal control. Data was analyzed using 2-way ANOVA. Statistical significance was determined by Tukey’s post-test. *PAX2* Fw: 5′-*GGCGAGAAGAGGAAACGTGA*-3′, *PAX2* Rv: 5′-*GAAGGTGCTTCCGCAAACTG-3*′, β*-actin Fw*: 5′-*CTCTGACTGACCGCGTTACT-3*′ and β*-actin* Rv: 5′-*TACCAACCATCACACCCTGAT-3*′.

## Results

### Chicken

**FIGURE 1:**
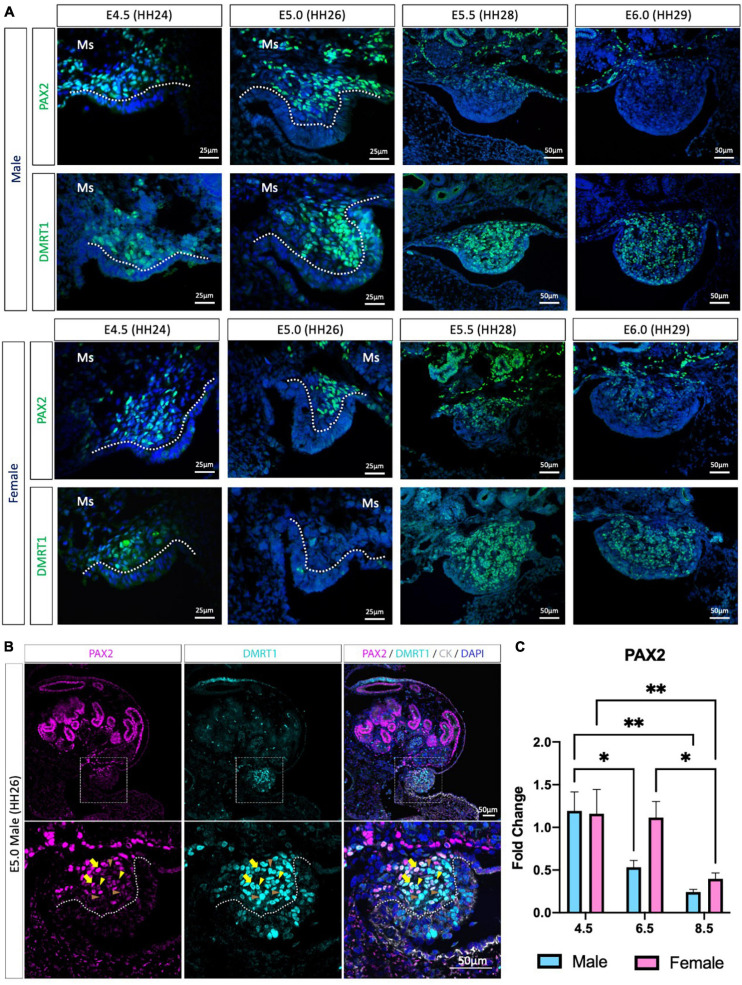
PAX2 expression in bipotential supporting cells before sex differentiation. **(A)** PAX2 and DMRT1 protein expression in E4.5, E5.0, E5.5, and E6.0 male and female chicken gonads. Dotted white line denotes the gonadal mesenchyme versus epithelium. Ms indicates the mesonephros. **(B)** PAX2 (magenta), DMRT1 (cyan), and cytokeratin (CK, white) immunofluorescence in E5.0 chicken urogenital system. Dashed white box indicates the magnification area; dotted white line denotes the gonadal mesenchyme versus epithelium. Yellow arrows show cells expressing both DMRT1 and PAX2 at high levels; yellow arrowheads indicate DMRT1^+^ cells expressing low levels of PAX2; brown arrowheads indicate DMRT1 positive PAX2 negative cells. **(C)** Decline in *PAX2* mRNA expression during gonadal sex differentiation. *PAX2* mRNA expression by qRT-PCR in E4.5, E6.5, and E8.5 male and female gonads. Expression level is relative to β-actin and normalized to E4.5 male. Bars represent Mean ± SEM. * and ** = adjusted *p* value < 0.05 and <0.01, respectively. 2-way ANOVA and Tukey’s post-test.

Previous chicken single-cell RNA-seq identified the gonadal supporting cell precursors as a mesenchymal population expressing the transcription factors *PAX2, DMRT1, OSR1* and the signaling molecule, *WNT4* (Estermann et al., 2020). PAX2 and DMRT1 immunofluorescence was performed to evaluate PAX2 expression pattern before, during and after gonadal differentiation (Figure 1 and Supplementary Figure 1). At E4.5 (HH24) and E5.0 (HH26), PAX2 positive cells were detected in the gonadal medulla in both sexes (Figure 1A). In males, from E5.5 (HH28) to E6.0 (HH29), PAX2 expression continued to be present in the basal region of the gonad but was absent in the most apical (Figure 1A). In females, a similar pattern occurred at E6.0 (Figure 1A). This data suggests that PAX2 is down-regulated at the onset of gonadal sex differentiation.

We previously found that *PAX2* mRNA was co-expressed with DMRT1 protein in gonadal sections (Estermann et al., 2020), but the co-localization of PAX2 and DMRT1 proteins in the same cells/nucleus was not assessed, because both primary antibodies were raised in rabbit. Iterative indirect immunofluorescence imaging (4i) (Gut et al., 2018) was used here to detect DMRT1 and PAX2 proteins in the same undifferentiated chicken gonad just prior to sexual differentiation at E5.0 (HH26) (Figure 1B and Supplementary Figure 1A). PAX2 positive cells in the gonadal mesenchyme were also DMRT1 positive. Interestingly, a gradient of PAX2 expression was noted; high in some cells (basal), lower in others (apical). This presumably reflects the gene being down-regulated among cells. Additionally, some DMRT1 positive cells were negative for PAX2, as expected, due to DMRT1 being expressed in the left coelomic epithelium and in the germ cells.

To evaluate the expression pattern of PAX2 in differentiating embryonic chicken gonads, immunofluorescence was performed on E6.5 (HH30) and E8.5 (HH34) male and female gonads (Supplementary Figures 1B–D). PAX2 was not expressed in E6.5 testis (Supplementary Figure 1B), consistent with previous reports of PAX2 down-regulation upon sexual differentiation (Estermann et al., 2020). DMRT1, AMH, and SOX9 immunofluorescence confirmed that these gonads were presumptive testes (Supplementary Figure 1B). In females, PAX2 expression was absent from the apical region of the gonad, whereas it was still expressed in the basal region of the gonad at E6.5 (Supplementary Figure 1C). This expression pattern was complementary to FOXL2 expression pattern, more strongly expressed in the gonadal apical than the basal region (Supplementary Figure 1C). Only few aromatase positive cells were detected in the gonad at this stage, suggesting that the gonadal differentiation had just commenced. As for the male, the female gonad also showed PAX2 being down-regulated after sex differentiation, on a cell-to-cell basis. By E8.5, PAX2 expression was excluded from the gonad in both sexes (Supplementary Figure 1D). To quantify these changes in *PAX2* expression, qRT-PCR was performed in male and female gonads at E4.5 (HH24), E6.5 (HH30) and E8.5 (HH34). These time points correspond to the period before, at the onset and after the onset of morphological gonadal sex differentiation. Consistent with the immunofluorescence data, *PAX2* expression was significantly reduced after gonadal sex differentiation in both sexes (E6.5 and E8.5) (Figure 1C). In females, expression reduction was delayed, occurring by E8.5 (Figure 1C). This suggests that male gonad sex differentiation commences prior to female gonadal sex differentiation. In conclusion, PAX2 is expressed in chicken undifferentiated gonadal supporting cells, co-localizing with DMRT1 in the medulla, and its expression is down-regulated during sexual differentiation.

### Quail

**FIGURE 2:**
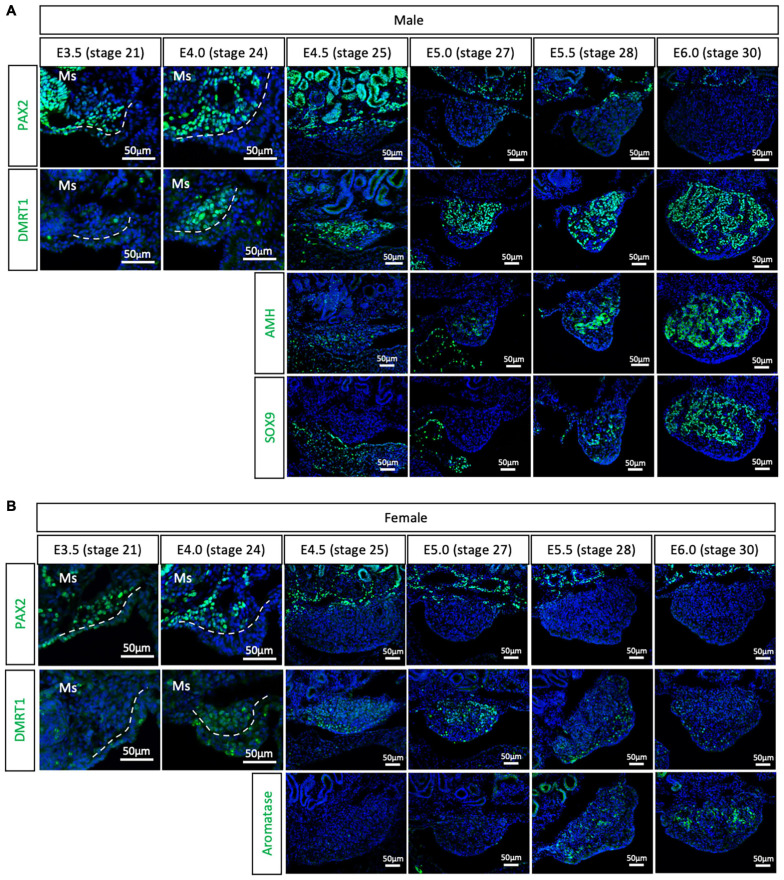
PAX2^+^ mesenchymal origin of supporting cells is conserved in quails. **(A)** PAX2, DMRT1, AMH, and SOX9 protein expression in E3.5, E4.0, E4.5, E5.0, E5.5, and E6.0 male quail gonads. **(B)** PAX2, DMRT1, and Aromatase protein expression in E3.5, E4.0, E4.5, E5.0, E5.5, and E6.0 female quail gonads. Dashed white line denotes the gonadal epithelial versus medullary mesenchyme. Ms indicates the mesonephros. DAPI was used as counterstain.

To evaluate if the PAX2^+^ mesenchymal origin of gonadal supporting cells is conserved among birds or is specific to chicken, gonads were analyzed from all three main bird clades. The Japanese quail (*Coturnix japonica*) belongs to the Galloanserae clade, the same group as chicken. This means that gonadal differentiation is likely to be very conserved between the two species. All previous studies on quail gonadal sex differentiation have relied upon histology to define the onset of sexual differentiation. Consequently, the timing of gonadal sex differentiation in this species has been variably reported, from between E6 (Kannankeril and Domm, 1968), E7 (Mohamed et al., 2017), and E8 (Intarapat and Satayalai, 2014). Quail gonadal sex differentiation was analyzed by immunofluorescence from E3.5 (stage 21) to E6.0 (stage 30), in half day incubation intervals (Figure 2). PAX2 was used as a (presumed) undifferentiated supporting cell precursor marker. Meanwhile, DMRT1, SOX9 and AMH were used as Sertoli cell markers in the testis and aromatase as pre-granulosa cell marker in developing ovary. Consistent with the chicken data, PAX2 positive cells started colonizing the region underlying the coelomic epithelium in both sexes of quail gonads at E3.5 (stage 21) and E4.0 (stage 24) (Figure 2). Unlike in chicken, DMRT1 was not detected in the undifferentiated quail gonads. Instead, by E4.0, DMRT1 expression was first detected and already sexually dimorphic, showing higher intensity levels in males than females (Figures 2A,B). By E4.5, PAX2 expression was turned off in the gonadal cells and its expression was excluded from the gonad during subsequent time points, when DMRT1 and other markers of sexual differentiation were activated (Figure 2). Some PAX2 positive cells were still visible in the basal region of the gonad, adjacent to the mesonephros (Figure 2). In addition, higher DMRT1 expression in males also suggests that the supporting cells commenced differentiation into Sertoli cells (Figure 2A). Some AMH positive cells were observed in the gonadal mesenchyme at E4.5 and E5.0 (stage 27) in both male and female gonads, but no SOX9 (Sertoli cell marker) (Figure 2A) or aromatase (pre-granulosa cell marker) (Figure 2B). The latter two were detected from E5.5 (stage 28). This quail data indicates that the PAX2^+^ mesenchymal origin of supporting cells observed in chicken is conserved among Galliformes. In addition, PAX2 down-regulation indicates that gonads commence gonadal sex differentiating at E4.5 in quail, much earlier than reported. This indicates that gene expression is a better predictor than morphological markers in defining the precise onset of gonadal sex determination.

### Zebra Finch

**FIGURE 3:**
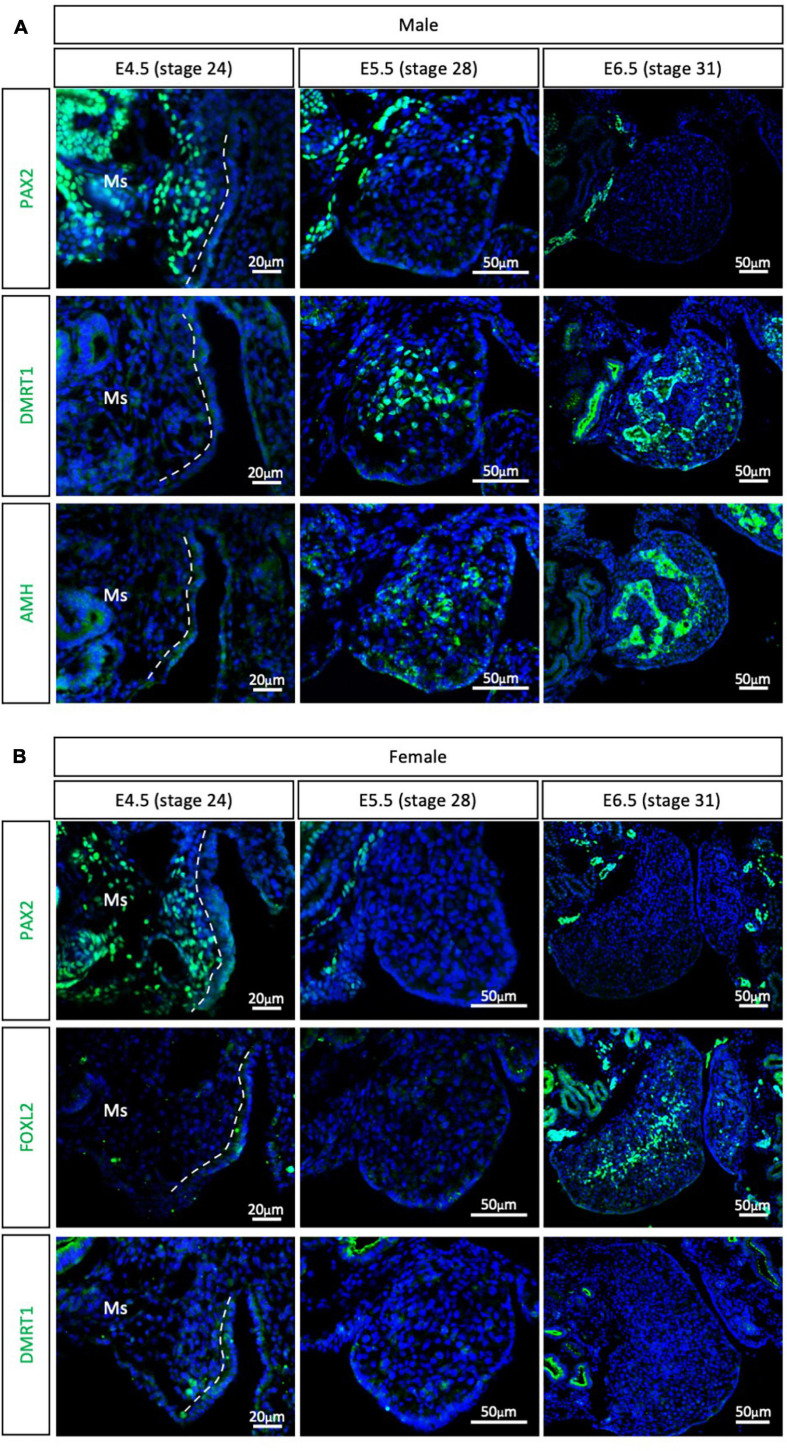
PAX2^+^ mesenchymal origin of supporting cells is conserved in Neoaves (zebra finch). **(A)** PAX2, DMRT1, and AMH protein expression E4.5, E5.5, and E6.5 male zebra finch gonads. **(B)** PAX2, FOXL2, and DMRT1 protein expression in E4.5, E5.0, E5.5, and E6.5 female zebra finch gonads. Dashed white line indicates the gonadal epithelial and mesenchyme limit. Ms indicates the mesonephros. DAPI was used as counterstain.

The other major clade of the Neoganthae is the Neoaves (perching birds). This group contains almost 95% of all living modern birds and is the result of early and rapid diversification around the Cretaceous mass extinction event (Claramunt and Cracraft, 2015; Prum et al., 2015). One of the most widely studied models in this clade is the zebra finch (*Taeniopygia guttata*), primarily in the field of neurobiology. Due to its popularity, the zebra finch genome was the second avian genome to be sequenced (Warren et al., 2010; Mak et al., 2015; Patterson and Fee, 2015). In addition, embryonic gonadal sex differentiation and primordial germ cell colonization have been studied in zebra finch, showing some differences between previous chicken reports (Hirst et al., 2017a; Jung et al., 2019). Zebra finch gonads have been reported to be sexually differentiated at E6.5, evidenced by *SOX9* and *FOXL2* mRNA expression in males and females, respectively (Hirst et al., 2017a). At E4.5 these markers are not expressed, suggesting an undifferentiated state (Hirst et al., 2017a). To evaluate if PAX2^+^ mesenchymal origin of supporting cells is conserved in Neoaves, PAX2, DMRT1, FOXL2, and AMH immunofluorescence was performed in male and female zebra finch gonads at E4.5 (stage 24), E5.5 (stage 28) and E6.5 (stage 31). PAX2 positive mesenchymal cells were detected in both male and female gonads at E4.5 (Figure 3). DMRT1, AMH and FOXL2 were not detected in the gonads at this stage. Altogether, this suggests that the zebra finch gonads are undifferentiated at E4.5. This is consistent with previous reports (Hirst et al., 2017a).

By E5.5, PAX2 expression was extinguished from both male (Figure 3A) and female (Figure 3B) gonads. DMRT1 and AMH positive Sertoli cells were identified in the male testicular medulla at E5.5 (Figure 3A). The downregulation of PAX2 and up-regulation of supporting cell markers indicates that gonadal sex differentiation in zebra finches commences at E5.5. By E6.5, FOXL2 expression was detected in the ovarian medulla (Figure 3B). In males, DMRT1 and AMH positive testicular cords were evident in the gonadal medulla (Figure 3A). Altogether, this data confirms the conservation of PAX2^+^ mesenchymal cell origin of supporting cells in Neoaves, and in particular in zebra finch. In addition, using PAX2 as a predictor of sex differentiation we were able to determine that zebra finch gonadal sex differentiation begins at E5.5 (stage 28).

### Emu

**FIGURE 4:**
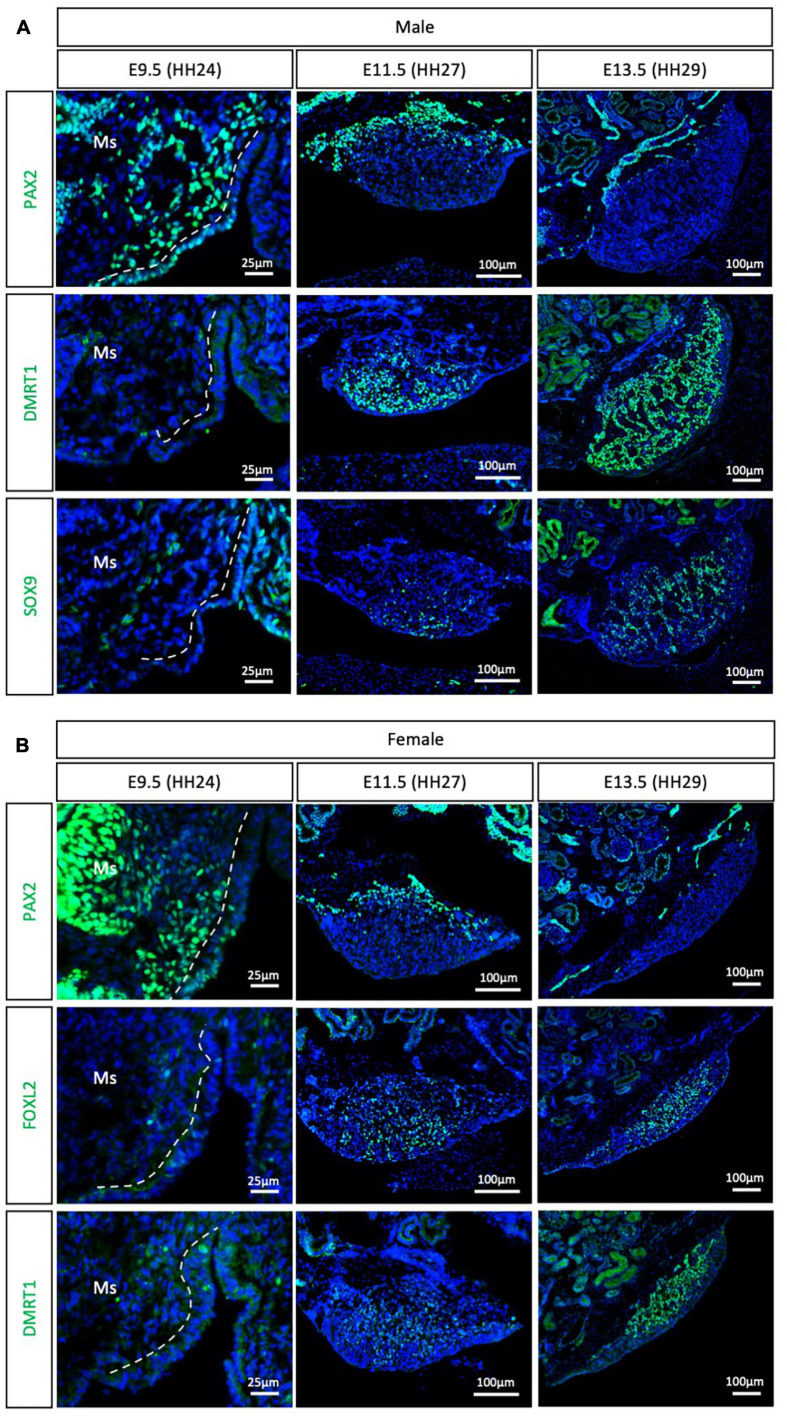
PAX2^+^ mesenchymal origin of supporting cells is conserved in ratites (emu). **(A)** PAX2, DMRT1, and SOX9 protein expression in E9.5, E11.5, and E13.5 male emu gonads. **(B)** PAX2, FOXL2, and DMRT1 protein expression in E9.5, E11.5, and E13.5 female emu gonads. Dashed white line indicates the gonadal epithelium vs. medullary mesenchyme. Ms indicates the mesonephros. DAPI was used as counterstain.

The Palaeognathae superorder includes the flightless ratites and the volant neotropical tinamou. Among the ratites, gonadal sex differentiation has only been described in the emu (*Dromaius novaehollandiae*) (Hirst et al., 2017a). Previous histological data suggested that emu gonadal differentiation commences at E16, evidenced by the presence of seminiferous cords in male gonads, containing DMRT1^+^ Sertoli cells (Hirst et al., 2017a). As noted previously, histological analysis is not the best methodology for defining the onset of sex differentiation. To gain insight into the specific timeframe of gonadal sex differentiation in emu and assess if PAX2 mesenchymal origin of supporting cells is conserved in ratites, gonadal immunofluorescence was performed at E9.5 (HH24), E11.5 (HH27) and E13.5 (HH29). PAX2 positive cells were detected at E9.5 in the medullary mesenchyme of both male and female emu gonads (Figure 4). This expression pattern is similar to the previous data shown for chicken, quail and zebra finch. FOXL2, SOX9 and DMRT1 were not expressed at E9.5 (HH24) (Figure 4), indicating that the gonads were undifferentiated and bipotential. By E11.5 (HH27), PAX2 expression was extinguished from the gonad in both sexes. In males DMRT1 and, to a lesser extent, SOX9, were expressed in the E11.5 (HH27) testis, indicating activation of the testicular differentiation pathway (Figure 4A). Similarly, in females, FOXL2 expression was up-regulated, indicating that the ovarian differentiation program had commenced (Figure 4B). By E13.5, in males, DMRT1^+^/SOX9^+^ testicular cords were identified in the gonadal medulla (Figure 4A). In females, FOXL2 was expressed in pre-granulosa cells in the ovarian medulla (Figure 4B). This data indicates that emu gonadal sex differentiation commences at E11.5 (HH27), earlier than previous reports based on histology. In addition, a PAX2 + mesenchymal origin of supporting cells is also conserved in the Palaeognathae clade.

## Discussion

The data presented here support a conserved origin of gonadal supporting cells in birds, distinct from that reported in the mouse. In the mouse model, the supporting cell lineage derives from the coelomic epithelium (Stevant et al., 2018, 2019). The gonadal supporting cells in birds do not derive from the coelomic epithelium but rather from a *DMRT1* and *PAX2* positive mesenchymal population. In this study, we show that PAX2 is expressed in the bipotential supporting cells of the gonadal mesenchyme in members of the Galloanserae (chicken and quail), Neoaves (zebra finch) and Paleognathae (emu), suggesting a conserved mechanism among all birds. In addition, this is the first systematic evaluation of gonadal sex differentiation in quail, emu and zebra finch using expression of gonadal marker proteins.

Previous reports based in histological and morphological analysis of the quail gonad have not consistently determined an embryonic stage of gonadal sex differentiation. Previous estimates of gonadal sex differentiation onset ranged from E5.5 to E8.0 (Kannankeril and Domm, 1968; Intarapat and Satayalai, 2014; Mohamed et al., 2017). The results reported here indicate that quail gonad sex differentiation commences at E4.5, earlier than previously suggested. This is shown by down-regulation of the undifferentiated supporting cell marker PAX2 and the up-regulation of DMRT1 in male gonads (Figure 2). These results show that gene expression analysis is more accurate than morphological and histological analysis in determining the onset of gonadal sex differentiation. Similarly, previous histological analysis of emu gonads suggested that sex differentiation commences at E16 (Hirst et al., 2017a). The data presented here indicates that emu gonadal sex determination commences at E11.5 (Figure 4), earlier than previous histological data suggests. In zebra finch, previous reports suggested the onset of sex differentiation occurs between E4.5 (undifferentiated) and E6.5 (differentiated). Our results agree with this data, showing that zebra finch gonadal sex differentiation commences at E5.5 (Figure 3). Down-regulation of PAX2 expression precisely predicted the onset of sexual differentiation in the three avian clades, more accurately than previous histological analysis. This research identifies PAX2 as a new marker for evaluating gonadal differentiation in birds.

In female chicken embryos, FOXL2 and aromatase proteins were expressed in the apical supporting cells at E6.5 (HH 30) (Supplementary Figure 1C), suggesting that those are the first supporting cells to differentiate. This apical-basal wave of differentiation is consistent with the concentration of PAX2 positive cells in the basal region of the gonad (Figures 1A–C). A similar pattern was observed in male quail gonads. At E5.5 (stage 28), SOX9 and AMH proteins were expressed in the apical testicular cords of the quail, suggesting that those are the first supporting cells to differentiate, and by E6.0 all Sertoli cells were SOX9, AMH and DMRT1 positive (Figure 2A). Recently, two transcriptionally distinctive Sertoli cell populations in E10.5 chicken testis were identified. One expressed lower levels of *SOX9* and *DMRT1* and higher levels of *CBR4* and *GSTA2* and was located in the peripheral testicular cords. The second population was located in the basal region, expressing higher levels of *SOX9* and *DMRT1* but no *GSTA2* (Estermann et al., 2020). This suggests that there may be two distinctive stages of Sertoli cell maturation, inner immature and outer mature populations. Mouse *Sry* is expressed as a wave across the male gonad (Larney et al., 2014), starting from the central region of the genital ridges, and then extending to cranial and caudal poles (Polanco and Koopman, 2007). 3D imaging approaches would be crucial to be understand how supporting cell differentiation occurs in birds and to understand if gonadal development also follows a longitudinal wave of differentiation, as in mouse.

Despite conservation of cell types, recent single-cell RNA-seq data from embryonic chicken gonads has shown that cell lineage specification in the gonad may also vary substantially between birds and mammals (Estermann et al., 2020). This research has shown that there are two main sources of gonadal cells; the coelomic epithelium and the mesonephric mesenchyme (Sekido and Lovell-Badge, 2007; Estermann et al., 2020). The current study confirms that this is the case for members of all three major bird clades. It is unclear why birds exhibit this different developmental origin of the key supporting cell lineage. A mesenchymal origin of supporting cells could be an ancestral feature, lost in mammals, or a feature acquired by the avian lineage. Further research is required to evaluate if the mesenchymal origin of supporting cells also occurs among reptiles, amphibians or fish. A key difference between placental mammals and birds is the genetic sex-determination system (XY vs. ZW). It would be interesting to evaluate if the supporting cell origin is correlated with the genetic sex-determination system (XY vs. ZW), sex determining genes (Sry vs. DMRT1 vs. others) and if it is also present in environmental sex determining species. Reptiles have diverse sex determining systems, ranging from pure GSD with either XX/XY or ZZ/ZW sex chromosome systems, to GSD modifiable by egg temperature, through to complete temperature dependent sex determination (TSD) (Warner, 2011; Ge et al., 2017; Whiteley et al., 2021). In at least one turtle species with TSD, the supporting cell lineage has been shown to derive form the coelomic epithelium (Yao et al., 2004). At present, there is not observable correlation between the type of sex determining system and the sources of gonadal supporting cells. Gonadal epithelium lineage tracing by GFP electroporation is feasible in oviparous reptiles, which would shed light on the origin of gonadal cell lineages in these groups (Hirst et al., 2017b). Among reptiles, crocodilians are the closest living clade to birds (Green et al., 2014), making them an ideal model to test the possible synapomorphy of gonadal PAX2 in birds and to study the evolution of the mesenchymal epithelial supporting cell origin. A previous ultrastructural study suggested that the supporting cell lineage in the American alligator (*Alligator mississippiensis*) may be of coelomic epithelial origin (Smith and Joss, 1993).

In summary, this study demonstrates a conserved gonadal PAX2 positive mesenchymal expression pattern in representatives of all three bird clades. Analysis should be expanded to other avian species to evaluate the degree of conservation among birds more broadly. Among the Galloanserae, only Galliformes have been studied in any detail (chicken and quail). Anseriformes (ducks, geese, and swans) could be examined. In addition, among the Neovaes, only the zebra finch has been studied in any detail. During the rapid diversification that characterize the Neoaves, birds could exhibit other mechanisms of gonadal formation, diverging from the PAX2^+^ mesenchymal origin. It would be interesting to expand this study to more members of this diverse clade. Given the monophyly of birds and their conserved ZZ/ZW sex determining system, we postulate that the gonadal PAX2 mesenchymal expression pattern is prevalent among avians.

## Data Availability Statement

The original contributions presented in the study are included in the article/Supplementary Material, further inquiries can be directed to the corresponding author.

## Ethics Statement

The animal study was reviewed and approved by Animal Ethics Office, Monash University (AEC approval not required for avian embryos less than mid embryogenesis as per state legislation). The zebra finch colony is a captive population derived from wild caught birds under Deakin University Animal Ethics # G23-2018. The birds used in this study were several generation-captive birds derived from this initial population. As for emu, quail, and chicken, animal ethics was not required for the finch embryos, as they were harvested less than mid-gestation (permitted by Australian law).

## Author Contributions

ME designed and performed most of the experiments and analyzed the results. MM and JM contributed with additional experiments and analysis. AC and CS supervised the work. ME and CS wrote the manuscript. All authors read and approved the final manuscript.

## Conflict of Interest

The authors declare that the research was conducted in the absence of any commercial or financial relationships that could be construed as a potential conflict of interest.

## Publisher’s Note

All claims expressed in this article are solely those of the authors and do not necessarily represent those of their affiliated organizations, or those of the publisher, the editors and the reviewers. Any product that may be evaluated in this article, or claim that may be made by its manufacturer, is not guaranteed or endorsed by the publisher.

## References

[B1] Ainsworth S. J. Stanley R. L. Evans D. J. (2010). Developmental stages of the Japanese quail. *J. Anat.* 216 3–15. 10.1111/j.1469-7580.2009.01173.x 19929907 PMC2807971

[B2] Ayers K. L. Lambeth L. S. Davidson N. M. Sinclair A. H. Oshlack A. Smith C. A. (2015). Identification of candidate gonadal sex differentiation genes in the chicken embryo using RNA-seq. *BMC Genomics* 16:704. 10.1186/s12864-015-1886-5 26377738 PMC4574023

[B3] Bai D. P. Chen Y. Hu Y. Q. He W. F. Shi Y. Z. Fan Q. M. (2020). Transcriptome analysis of genes related to gonad differentiation and development in Muscovy ducks. *BMC Genomics* 21:438. 10.1186/s12864-020-06852-z 32590948 PMC7318502

[B4] Baillet A. Mandon-Pepin B. (2012). Mammalian ovary differentiation - a focus on female meiosis. *Mol. Cell. Endocrinol.* 356 13–23. 10.1016/j.mce.2011.09.029 21964319

[B5] Bowles J. Feng C. W. Spiller C. Davidson T. L. Jackson A. Koopman P. (2010). FGF9 suppresses meiosis and promotes male germ cell fate in mice. *Dev. Cell* 19 440–449. 10.1016/j.devcel.2010.08.010 20833365

[B6] Bowles J. Knight D. Smith C. Wilhelm D. Richman J. Mamiya S. (2006). Retinoid signaling determines germ cell fate in mice. *Science* 312 596–600. 10.1126/science.1125691 16574820

[B7] Carlon N. Stahl A. (1985). Origin of the somatic components in chick embryonic gonads. *Arch. Anat. Microsc. Morphol. Exp.* 74 52–59. 10.1007/bf00341521 4073912

[B8] Chassot A. A. Le Rolle M. Jolivet G. Stevant I. Guigonis J. M. Da Silva F. (2020). Retinoic acid synthesis by ALDH1A proteins is dispensable for meiosis initiation in the mouse fetal ovary. *Sci. Adv.* 6:eaaz1261. 10.1126/sciadv.aaz1261 32494737 PMC7244317

[B9] Claramunt S. Cracraft J. (2015). A new time tree reveals Earth history’s imprint on the evolution of modern birds. *Sci. Adv.* 1:e1501005. 10.1126/sciadv.1501005 26824065 PMC4730849

[B10] Clinton M. Haines L. Belloir B. Mcbride D. (2001). Sexing chick embryos: a rapid and simple protocol. *Br. Poult. Sci.* 42 134–138. 10.1080/713655025 11337963

[B11] Cracraft J. (2001). Avian evolution. Gondwana biogeography and the Cretaceous-Tertiary mass extinction event. *Proc. Biol. Sci.* 268 459–469. 10.1098/rspb.2000.1368 11296857 PMC1088628

[B12] DeFalco T. Capel B. (2009). Gonad morphogenesis in vertebrates: divergent means to a convergent end. *Annu. Rev. Cell Dev. Biol.* 25 457–482. 10.1146/annurev.cellbio.042308.13350 19807280 PMC4507502

[B13] Dickens M. J. Balthazart J. Cornil C. A. (2012). Brain aromatase and circulating corticosterone are rapidly regulated by combined acute stress and sexual interaction in a sex-specific manner. *J. Neuroendocrinol.* 24 1322–1334. 10.1111/j.1365-2826.2012.02340.x 22612582 PMC3510384

[B14] Eggers S. Ohnesorg T. Sinclair A. (2014). Genetic regulation of mammalian gonad development. *Nat. Rev. Endocrinol.* 10 673–683. 10.1038/nrendo.2014.163 25246082

[B15] Estermann M. A. Williams S. Hirst C. E. Roly Z. Y. Serralbo O. Adhikari D. (2020). Insights into gonadal sex differentiation provided by single-cell transcriptomics in the chicken embryo. *Cell Rep.* 31:107491. 10.1016/j.celrep.2020.03.055 32268081

[B16] Ge C. Ye J. Zhang H. Zhang Y. Sun W. Sang Y. (2017). Dmrt1 induces the male pathway in a turtle species with temperature-dependent sex determination. *Development* 144 2222–2233. 28506988 10.1242/dev.152033

[B17] Goodfellow P. N. Lovell-Badge R. (1993). SRY and sex determination in mammals. *Annu. Rev. Genet.* 27 71–92. 10.1146/annurev.ge.27.120193.000443 8122913

[B18] Green R. E. Braun E. L. Armstrong J. Earl D. Nguyen N. Hickey G. (2014). Three crocodilian genomes reveal ancestral patterns of evolution among archosaurs. *Science* 346:1254449. 10.1126/science.1254449 PMC4386873 25504731

[B19] Gut G. Herrmann M. D. Pelkmans L. (2018). Multiplexed protein maps link subcellular organization to cellular states. *Science* 361:eaar7042. 10.1126/science.aar7042 30072512

[B20] Hackett S. J. Kimball R. T. Reddy S. Bowie R. C. Braun E. L. Braun M. J. (2008). A phylogenomic study of birds reveals their evolutionary history. *Science* 320 1763–1768. 10.1126/science.1157704 18583609

[B21] Hamburger V. Hamilton H. L. (1951). A series of normal stages in the development of the chick embryo. *J. Morphol.* 88 49–92. 10.1002/jmor.1050880104 24539719

[B22] Handley L. J. Ceplitis H. Ellegren H. (2004). Evolutionary strata on the chicken Z chromosome: implications for sex chromosome evolution. *Genetics* 167 367–376. 10.1534/genetics.167.1.367 15166161 PMC1470863

[B23] Hirst C. E. Major A. T. Ayers K. L. Brown R. J. Mariette M. Sackton T. B. (2017a). Sex reversal and comparative data undermine the W chromosome and support Z-linked DMRT1 as the regulator of gonadal sex differentiation in birds. *Endocrinology* 158 2970–2987. 10.1210/en.2017-00316 28911174

[B24] Hirst C. E. Serralbo O. Ayers K. L. Roeszler K. N. Smith C. A. (2017b). “Genetic manipulation of the avian urogenital system using in ovo electroporation,” in *Avian and Reptilian Developmental Biology: Methods and Protocols*, ed. Sheng G. (New York, NY: Springer New York). 10.1007/978-1-4939-7216-6_11 28809021

[B25] Huynen L. Millar C. D. Lambert D. M. (2002). A DNA test to sex ratite birds. *Mol. Ecol.* 11 851–856. 10.1046/j.1365-294x.2002.01483.x 11972770

[B26] Intarapat S. Satayalai O. (2014). Microanatomical study of embryonic gonadal development in Japanese quail (*Coturnix japonica*). *Anat. Res. Int.* 2014:168614. 10.1155/2014/168614 PMC4168037 25276431

[B27] Ioannidis J. Taylor G. Zhao D. Liu L. Idoko-Akoh A. Gong D. (2021). Primary sex determination in birds depends on DMRT1 dosage, but gonadal sex does not determine adult secondary sex characteristics. *Proc. Natl. Acad. Sci. U. S. A.* 118:e2020909118. 10.1073/pnas.2020909118 33658372 PMC7958228

[B28] Jung K. M. Kim Y. M. Keyte A. L. Biegler M. T. Rengaraj D. Lee H. J. (2019). Identification and characterization of primordial germ cells in a vocal learning Neoaves species, the zebra finch. *FASEB J.* 33 13825–13836. 10.1096/fj.201900760rr 31604057

[B29] Kannankeril J. V. Domm L. V. (1968). Development of the gonads in the female Japanese quail. *Am. J. Anat.* 123 131–146. 10.1002/aja.1001230106 5702209

[B30] Koopman P. Gubbay J. Vivian N. Goodfellow P. Lovell-Badge R. (1991). Male development of chromosomally female mice transgenic for Sry. *Nature* 351 117–121. 10.1038/351117a0 2030730

[B31] Koopman P. Munsterberg A. Capel B. Vivian N. Lovell-Badge R. (1990). Expression of a candidate sex-determining gene during mouse testis differentiation. *Nature* 348 450–452. 10.1038/348450a0 2247150

[B32] Koubova J. Menke D. B. Zhou Q. Capel B. Griswold M. D. Page D. C. (2006). Retinoic acid regulates sex-specific timing of meiotic initiation in mice. *Proc. Natl. Acad. Sci. U. S. A.* 103 2474–2479. 10.1073/pnas.0510813103 16461896 PMC1413806

[B33] Lambeth L. S. Cummins D. M. Doran T. J. Sinclair A. H. Smith C. A. (2013). Overexpression of aromatase alone is sufficient for ovarian development in genetically male chicken embryos. *PloS One* 8:e68362. 10.1371/journal.pone.0068362 PMC3695963 23840850

[B34] Lambeth L. S. Raymond C. S. Roeszler K. N. Kuroiwa A. Nakata T. Zarkower D. (2014). Over-expression of DMRT1 induces the male pathway in embryonic chicken gonads. *Dev. Biol.* 389 160–172. 10.1016/j.ydbio.2014.02.012 24576538 PMC4201592

[B35] Larney C. Bailey T. L. Koopman P. (2014). Switching on sex: transcriptional regulation of the testis-determining gene *Sry*. *Development* 141 2195–2205. 10.1242/dev.107052 24866114 PMC4034426

[B36] Lawson K. A. (1999). Fate mapping the mouse embryo. *Int. J. Dev. Biol.* 43 773–775. 10668985

[B37] Lin Y. T. Barske L. Defalco T. Capel B. (2017). Numb regulates somatic cell lineage commitment during early gonadogenesis in mice. *Development* 144 1607–1618. 28360133 10.1242/dev.149203 PMC5450849

[B38] Liu C. F. Liu C. Yao H. H. (2010). Building pathways for ovary organogenesis in the mouse embryo. *Curr. Top. Dev. Biol.* 90 263–290. 10.1016/s0070-2153(10)90007-0 20691852 PMC3400115

[B39] Liu C. Rodriguez K. Yao H. H. (2016). Mapping lineage progression of somatic progenitor cells in the mouse fetal testis. *Development* 143 3700–3710. 27621062 10.1242/dev.135756 PMC5087644

[B40] Major A. T. Ayers K. Chue J. Roeszler K. Smith C. (2019). FOXL2 antagonises the male developmental pathway in embryonic chicken gonads. *J. Endocrinol.* 10.1530/JOE-19-0277 [Epub ahead of print]. 31505465

[B41] Mak S. S. Wrabel A. Nagai H. Ladher R. K. Sheng G. (2015). Zebra finch as a developmental model. *Genesis* 53 669–677. 10.1002/dvg.22900 26385755

[B42] Marshall Graves J. A. (2008). Weird animal genomes and the evolution of vertebrate sex and sex chromosomes. *Annu. Rev. Genet.* 42 565–586. 10.1146/annurev.genet.42.110807.091714 18983263

[B43] Mohamed G. Selim A. Abd-Elhafeez H. Mohamed M. (2017). Histomorphological developmental studies of the left ovary in the Japanese quail (*Coturnix Coturnix Japonica*). *Mathews J. Cytol. Histol.* 1:002.

[B44] Moreau J. L. M. Kesteven S. Martin E. Lau K. S. Yam M. X. O’reilly V. C. (2019). Gene-environment interaction impacts on heart development and embryo survival. *Development* 146:dev172957. 10.1242/dev.172957 30787001

[B45] Murray J. R. Varian-Ramos C. W. Welch Z. S. Saha M. S. (2013). Embryological staging of the Zebra Finch, *Taeniopygia guttata*. *J. Morphol.* 274 1090–1110. 10.1002/jmor.20165 23813920 PMC4239009

[B46] Nagai H. Mak S. S. Weng W. Nakaya Y. Ladher R. Sheng G. (2011). Embryonic development of the emu, *Dromaius novaehollandiae*. *Dev. Dyn.* 240 162–175. 10.1002/dvdy.22520 21181941

[B47] Nef S. Stevant I. Greenfield A. (2019). Characterizing the bipotential mammalian gonad. *Curr. Top. Dev. Biol.* 134 167–194. 10.1016/bs.ctdb.2019.01.002 30999975

[B48] Okuno M. Miyamoto S. Itoh T. Seki M. Suzuki Y. Mizushima S. (2020). Expression profiling of sexually dimorphic genes in the Japanese quail, *Coturnix japonica*. *Sci. Rep.* 10:20073. 10.1038/s41598-020-77094-y PMC7705726 33257723

[B49] Patterson M. M. Fee M. S. (2015). “Zebra finches in biomedical research,” in *Laboratory Animal Medicine*, eds Fox J. G. Anderson L. C. Otto G. M. Pritchett-Corning K. R. Whary M. T. (Boston: Academic Press). 10.1016/B978-0-12-409527-4.00023-7

[B50] Pfaffl M. W. (2001). A new mathematical model for relative quantification in real-time RT-PCR. *Nucleic Acids. Res.* 29:e45. 10.1093/nar/29.9.e45 11328886 PMC55695

[B51] Piprek R. P. Kloc M. Kubiak J. Z. (2016). “Early development of the gonads: origin and differentiation of the somatic cells of the genital ridges,” in *Molecular Mechanisms of Cell Differentiation in Gonad Development*, ed. Piprek R. P. (Cham: Springer International Publishing). 10.1007/978-3-319-31973-5_1 27300173

[B52] Piprek R. P. Kolasa M. Podkowa D. Kloc M. Kubiak J. Z. (2017). Cell adhesion molecules expression pattern indicates that somatic cells arbitrate gonadal sex of differentiating bipotential fetal mouse gonad. *Mech. Dev.* 147 17–27. 10.1016/j.mod.2017.07.001 28760667

[B53] Polanco J. C. Koopman P. (2007). *Sry* and the hesitant beginnings of male development. *Dev. Biol.* 302 13–24. 10.1016/j.ydbio.2006.08.049 16996051

[B54] Prum R. O. Berv J. S. Dornburg A. Field D. J. Townsend J. P. Lemmon E. M. (2015). A comprehensive phylogeny of birds (Aves) using targeted next-generation DNA sequencing. *Nature* 526 569–573. 26444237 10.1038/nature15697

[B55] Raymond C. S. Kettlewell J. R. Hirsch B. Bardwell V. J. Zarkower D. (1999). Expression of Dmrt1 in the genital ridge of mouse and chicken embryos suggests a role in vertebrate sexual development. *Dev. Biol.* 215 208–220. 10.1006/dbio.1999.9461 10545231

[B56] Rebourcet D. O’shaughnessy P. J. Pitetti J. L. Monteiro A. O’hara L. Milne L. (2014). Sertoli cells control peritubular myoid cell fate and support adult Leydig cell development in the prepubertal testis. *Development* 141 2139–2149. 10.1242/dev.107029 24803659 PMC4011090

[B57] Rotgers E. Jorgensen A. Yao H. H. (2018). At the crossroads of fate-somatic cell lineage specification in the fetal gonad. *Endocr. Rev.* 39 739–759. 10.1210/er.2018-00010 29771299 PMC6173476

[B58] Schneider C. A. Rasband W. S. Eliceiri K. W. (2012). NIH Image to ImageJ: 25 years of image analysis. *Nat. Methods* 9 671–675. 22930834 10.1038/nmeth.2089 PMC5554542

[B59] Sekido R. Lovell-Badge R. (2007). Mechanisms of gonadal morphogenesis are not conserved between chick and mouse. *Dev. Biol.* 302 132–142. 17026980 10.1016/j.ydbio.2006.09.007

[B60] Sinclair A. H. Berta P. Palmer M. S. Hawkins J. R. Griffiths B. L. Smith M. J. (1990). A gene from the human sex-determining region encodes a protein with homology to a conserved DNA-binding motif. *Nature* 346 240–244. 10.1038/nmeth.2089 1695712

[B61] Smith C. A. Joss J. M. P. (1993). Gonadal sex differentiation in *Alligator mississippiensis*, a species with temperature-dependent sex determination. *Cell Tissue Res.* 273 149–162. 10.1186/s12864-016-2396-9 26810479 PMC4727388

[B62] Smith C. A. Sinclair A. H. (2004). Sex determination: insights from the chicken. *Bioessays* 26 120–132. 10.1002/bies.10400 14745830

[B63] Smith C. A. Katz M. Sinclair A. H. (2003). DMRT1 is upregulated in the gonads during female-to-male sex reversal in Zw chicken embryos. *Biol. Reprod.* 68 560–570. 10.1095/biolreprod.102.007294 12533420

[B64] Smith C. A. Roeszler K. N. Ohnesorg T. Cummins D. M. Farlie P. G. Doran T. J. (2009). The avian Z-linked gene DMRT1 is required for male sex determination in the chicken. *Nature* 461 267–271. 10.1038/nature08298 19710650

[B65] Soderstrom K. Qin W. Leggett M. H. (2007). A minimally invasive procedure for sexing young zebra finches. *J. Neurosci. Methods* 164 116–119. 10.1016/j.jneumeth.2007.04.007 17532050 PMC2350111

[B66] Spiller C. Koopman P. Bowles J. (2017). Sex determination in the mammalian germline. *Annu. Rev. Genet.* 51 265–285. 10.1146/annurev-genet-120215-035449 28853925

[B67] Stevant I. Nef S. (2019). Genetic control of gonadal sex determination and development. *Trends Genet.* 35 346–358. 10.1016/j.tig.2019.02.004 30902461

[B68] Stevant I. Kuhne F. Greenfield A. Chaboissier M. C. Dermitzakis E. T. Nef S. (2019). Dissecting cell lineage specification and sex fate determination in gonadal somatic cells using single-cell transcriptomics. *Cell Rep.* 2 3272–3283.e3. 10.1016/j.celrep.2019.02.069 30893600

[B69] Stevant I. Neirijnck Y. Borel C. Escoffier J. Smith L. B. Antonarakis S. E. (2018). Deciphering cell lineage specification during male sex determination with single-cell RNA sequencing. *Cell Rep.* 22 1589–1599. 10.1016/j.celrep.2018.01.043 29425512

[B70] Suzuki A. Saga Y. (2008). Nanos2 suppresses meiosis and promotes male germ cell differentiation. *Genes Dev.* 22 430–435. 18281459 10.1101/gad.1612708 PMC2238665

[B71] Tagami T. Miyahara D. Nakamura Y. (2017). Avian primordial germ cells. *Adv. Exp. Med. Biol.* 1001 1–18. 10.1016/j.celrep.2018.01.043 28980226

[B72] Takada S. Ota J. Kansaku N. Yamashita H. Izumi T. Ishikawa M. (2006). Nucleotide sequence and embryonic expression of quail and duck Sox9 genes. *Gen. Comp. Endocrinol.* 145 208–213. 10.1016/j.ygcen.2005.08.009 16216246

[B73] Vernet N. Condrea D. Mayere C. Feret B. Klopfenstein M. Magnant W. (2020). Meiosis occurs normally in the fetal ovary of mice lacking all retinoic acid receptors. *Sci. Adv.* 6:eaaz1139. 10.1126/sciadv.aaz1139 PMC7244263 32917583

[B74] Warner D. A. (2011). “Chapter 1 - Sex determination in reptiles,” in *Hormones and Reproduction of Vertebrates*, eds Norris D. O. Lopez K. H. (London: Academic Press).

[B75] Warren W. C. Clayton D. F. Ellegren H. Arnold A. P. Hillier L. W. Kunstner A. (2010). The genome of a songbird. *Nature* 464 757–762. 20360741 10.1038/nature08819 PMC3187626

[B76] Whiteley S. L. Holleley C. E. Wagner S. Blackburn J. Deveson I. W. Marshall Graves J. A. (2021). Two transcriptionally distinct pathways drive female development in a reptile with both genetic and temperature dependent sex determination. *PLoS Genet.* 17:e1009465. 10.1371/journal.pgen.1009465 33857129 PMC8049264

[B77] Wilhelm D. Palmer S. Koopman P. (2007). Sex determination and gonadal development in mammals. *Physiol. Rev.* 87 1–28. 17237341 10.1152/physrev.00009.2006

[B78] Xu L. Auer G. Peona V. Suh A. Deng Y. Feng S. (2019). Dynamic evolutionary history and gene content of sex chromosomes across diverse songbirds. *Nat. Ecol. Evol.* 3 834–844. 30936435 10.1038/s41559-019-0850-1

[B79] Yang Y. Workman S. Wilson M. (2018). The molecular pathways underlying early gonadal development. *J. Mol. Endocrinol*. 10.1530/JME-17-0314 [Epub ahead of print]. 30042122

[B80] Yao H. H. Dinapoli L. Capel B. (2004). Cellular mechanisms of sex determination in the red-eared slider turtle, *Trachemys scripta*. *Mech Dev* 121 1393–1401. 15454268 10.1016/j.mod.2004.06.001 PMC4067764

[B81] Yao H. H. Whoriskey W. Capel B. (2002). Desert Hedgehog/Patched 1 signaling specifies fetal Leydig cell fate in testis organogenesis. *Genes Dev.* 16 1433–1440. 12050120 10.1101/gad.981202 PMC186321

[B82] Zhou Q. Zhang J. Bachtrog D. An N. Huang Q. Jarvis E. D. (2014). Complex evolutionary trajectories of sex chromosomes across bird taxa. *Science* 346:1246338. 10.1126/science.1246338 PMC6445272 25504727

