# Supplementary material for: PAX2+ Mesenchymal Origin of Gonadal Supporting Cells Is Conserved in Birds
Source: Front Cell Dev Biol. 2021 Aug 27;9:735203. doi: 10.3389/fcell.2021.735203 (PMC8429852; doi:10.3389/fcell.2021.735203)
Supplement: Supplementary file 1 [file Data_Sheet_1.docx]

Supplementary Material


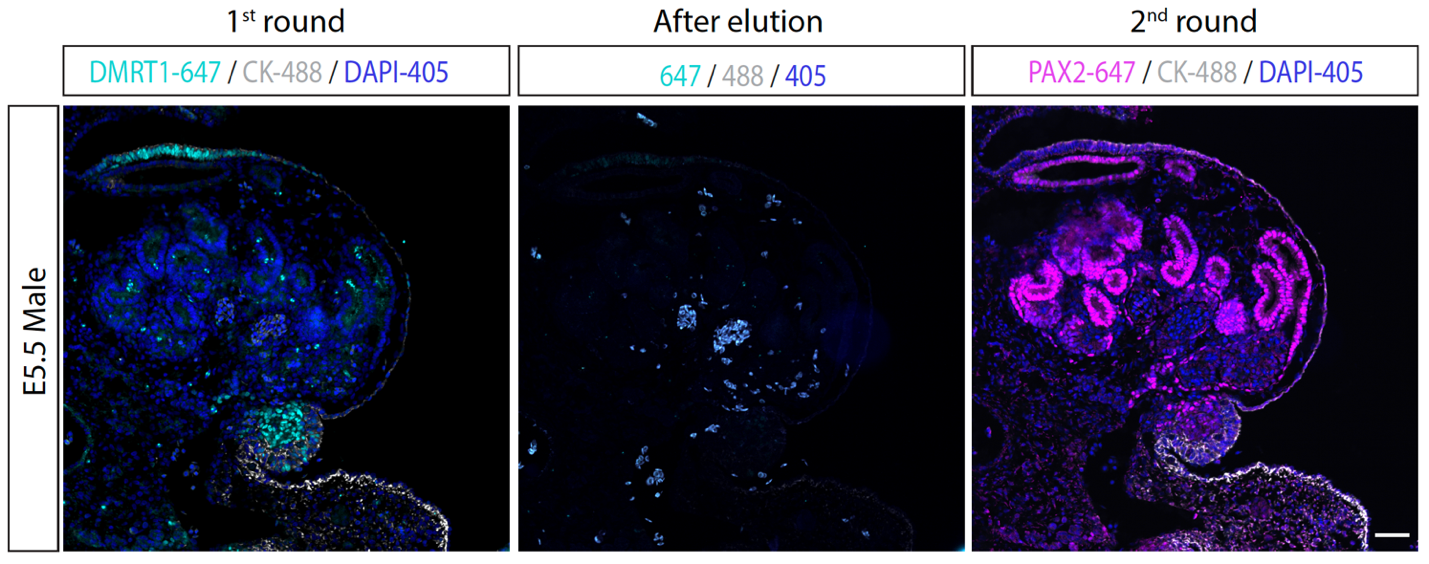

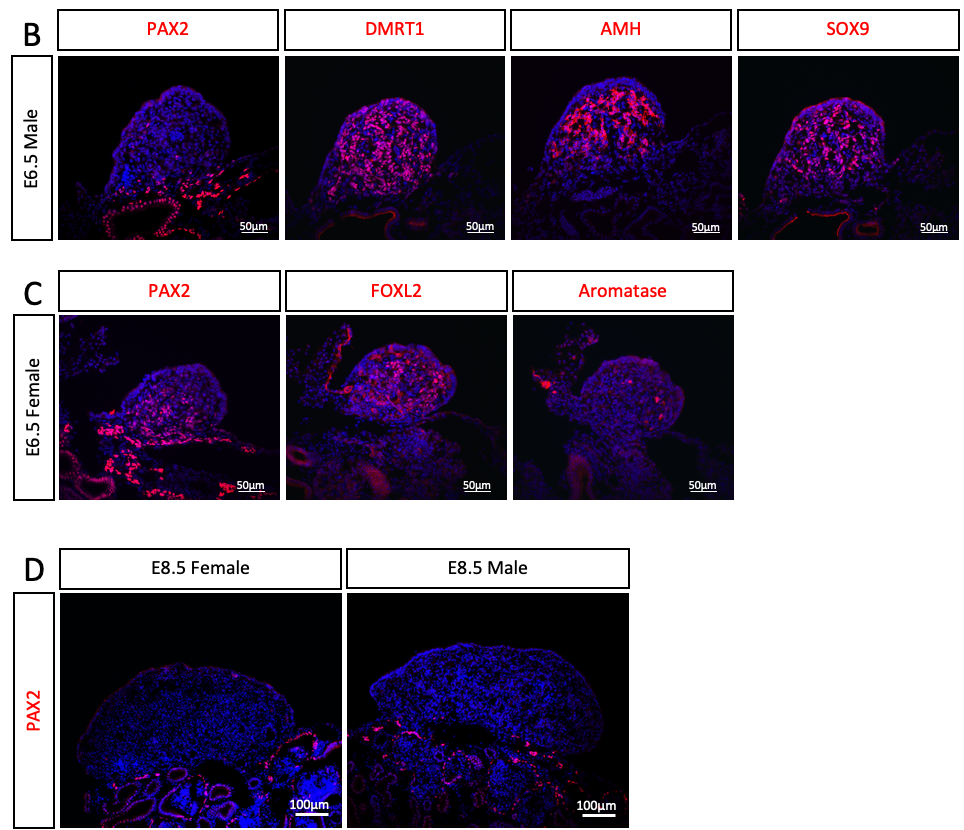


A

**Supplementary Figure 1.** PAX2 is downregulated in differentiating chicken gonads (A)PAX2 and DMRT1 immunostaining is specific. Following the 4i protocol, the section was first incubated with anti-DMRT1 (Cyan) and anti-Cytokeratin (CK, grey). After elution, the signals are undetectable. The same section was then re-stained with anti-PAX2 (magenta) and anti-CK (grey) in the same channels. (B) PAX2, DMRT1, AMH and SOX9 protein expression in E6.5 (HH30) male chicken gonads. (C) PAX2 FOXL2 and aromatase protein expression in E6.5 female chicken gonads. (D) Loss of PAX2 expression in male and female chicken gonads at E8.5 (HH35). DAPI was used as counterstain.
